# Current Social Perception of and Value Attached to Nursing Professionals’ Competences: An Integrative Review

**DOI:** 10.3390/ijerph19031817

**Published:** 2022-02-05

**Authors:** Margarita Rodríguez-Pérez, Francisco Mena-Navarro, Abraham Domínguez-Pichardo, Cristina Teresa-Morales

**Affiliations:** 1 Department of Nursing, University of Huelva, 21007 Huelva, Spain; margaro@denf.uhu.es (M.R.-P.); fjmena@denf.uhu.es (F.M.-N.); 2 Centro de Salud Bombero Etxaniz, Osakidetza, 48010 Bilbao, Spain; abrahampichardo15@gmail.com

**Keywords:** literature review, nursing, public image, social image, social perception, social representation

## Abstract

In order to develop nurses’ identities properly, they need to publicise their professional competences and make society aware of them. For that, this study was conducted to describe the competences that society currently attributes to nursing professionals and how nursing is valued in society. This review was based on the conceptual framework by Whittemore and Knafl. The literature search was conducted using PubMed, WOS, and CINAHL databases, and the search strategy was based on a combination of natural language and standardised keywords, with limits and criteria for inclusion, exclusion, and quality. The results of the studies were classified and coded in accordance with the competence groups of the professional profile described in the Tuning Educational Structures in Europe programme. Fourteen studies were selected. The most commonly reported competence groups were as follows: nursing practice and clinical decision making; and communication and interpersonal competences. Nursing is perceived as a healthcare profession dedicated to caring for individuals. Its other areas of competence and its capacity for leadership are not well known. In order to develop a professional identity, it is essential to raise awareness of the competences that make up this professional profile.

## 1. Introduction

A nurse’s professional identity (NPI) was described by Fargemoen as “the values and beliefs held by the nurse that guide her/his thinking, actions, and interaction with the patient” [1] (p. 437) that are considered to be inherent to professional development. The four key elements that make up NPI are the theoretical and practical knowledge that professional nurses must acquire; the definition of the professional role, setting out what nurses must know, do, think, and feel; the nurses’ own social and moral values, which cause them to behave in an expected, desirable manner; and the social image or representation of the profession, encompassing the prestige and value assigned to it by society [1,2,3].

Thus, the social or public image of the profession is a key component of professional identity. Generally speaking, nursing is recognised within society as a healthcare profession in its own right [4], whose essence lies in delivering care in close contact and developing ongoing relationships with patients, who are always vulnerable and completely or partially dependent [1]. The nursing profession is demanding, advocating for, and constructing a body of distinct, specific scientific knowledge that is produced and corroborated by its members [5]. This would provide a reasoned foundation for NPI to differentiate nursing from other professions and define its nature, characteristics, knowledge, and activities. However, it is very important that these aspects are made visible and highlighted within society so that the nursing profession is recognised for what it is. A number of studies highlight the fact that nursing professionals perceive patients as being unaware of and not understanding the role and tasks involved in nurses’ professional performance [6,7]. This lack of knowledge is even present among nursing students themselves, who, before starting the nursing degree, were not able to describe what nursing is or what nurses do [8,9,10]. As a result, nursing professionals are frequently mistaken for other healthcare workers [11,12,13] or defined by comparing healthcare workers with one other [14]. Therefore, it seems that the general public is unaware of the current academic, scientific, and professional situation of the profession, resulting in the profession often being misrepresented [4,15]. This may stem from the image that is sometimes portrayed in the media, the image of a secondary, passive, limited profession [6] that does not reflect its real competencies, thus rendering its competencies either invisible or unrecognised by society [16].

In the last decade, the nursing profession has evolved largely due to curriculum changes and the enactment of legislation aimed at structuring healthcare professions. In the United States, in 2021, the American Association of Colleges of Nurses established new guidelines for the purpose of shaping the education of nurses in a report entitled The Essentials: Core Competencies for Professional Nursing Education. This report lists 10 domains and their associated competencies, which are the essence of the nursing profession and practice [17]. In turn, in Europe, the Bologna declaration initiated a new competence-based training model developed through the Tuning Educational Structures in Europe programme in 1999 [18]. Both models describe curriculum outcomes in terms of competences, understood as the dynamic combination of attributes regarding knowledge, skills, attitudes, and responsibilities. As a result, nursing professions are defined by the set of competences that their professionals must possess. These programmes established the competencies that define the nursing profession by classifying them into five groups of competencies, as in the case of Europe, or into ten domains, as in the case of the United States [17,18]. Table 1 describes both classifications and identifies common areas between them.

This competence-based structure offers new guidance for understanding and learning nursing, and for current and future nursing professionals and students to work on their NPIs. It is therefore essential to gain an understanding of what is perceived about what nurses do and/or can do and the value that society places on the profession. Therefore, the aim of this study was to describe the competences that society currently attributes to nursing professionals and how nursing is valued in society.

## 2. Materials and Methods

Design: An integrative review was made. The stages followed in this review are based on the methodological framework developed by Whittemore and Knafl [19]: problem identification; rigorous search strategy; comprehensive evaluation; interpretation and critical analysis of selected data; synthesis and presentation of selected data.

Search Method: The databases explored were PubMed, WOS, and CINAHL. The search strategy was based on a combination of the following keywords: social perception/image/representation and nurse/nurses/nursing. The thesaurus of each database was selected for the search using standardised terms. Natural language keywords were used in the search by title and abstract. Details of the search strategies employed can be seen in Table 2. The search was conducted in April and May 2021. Finally, key authors in the selected documents were identified, and the snowballing technique was used.

Inclusion and exclusion criteria: The search was limited to studies published between 2016 and 2021 in English, Spanish, and/or Portuguese. The inclusion criteria were as follows: articles whose object of study was the social image of nursing (IC1); original or review articles using a qualitative, quantitative, and/or mixed methodology (IC2).The exclusion criteria were as follows: articles using ahistorical methodology (EC1); studies with samples of nursing students in their second year and above or samples of professional nurses (EC2).The latter criterion was applied because first-year nursing students’ social image of the nursing profession remains intact. However, over time, their social image becomes self-image and is influenced by academic training and clinical placements.

Quality appraisal: The methodological quality of the studies was assessed using MMAT [20], an appraisal tool for qualitative, quantitative, and mixed methods studies and reviews. The templates for the different methodologies were reviewed independently by the researchers. A low methodological quality score was used as an exclusion criterion (EC3).

Data extraction and analysis: The data extraction and synthesis followed the steps set out by Whittemore and Knafl [19]. The researchers read the selected studies exhaustively. Using an analytical comparative framework, the results of the studies were classified and coded in accordance with the competence groups defined by the Tuning Programme, as described in the Introduction section. We believe that this coding is flexible and specific enough to define the competence profile of a nurse in any context.

**Figure 1:**
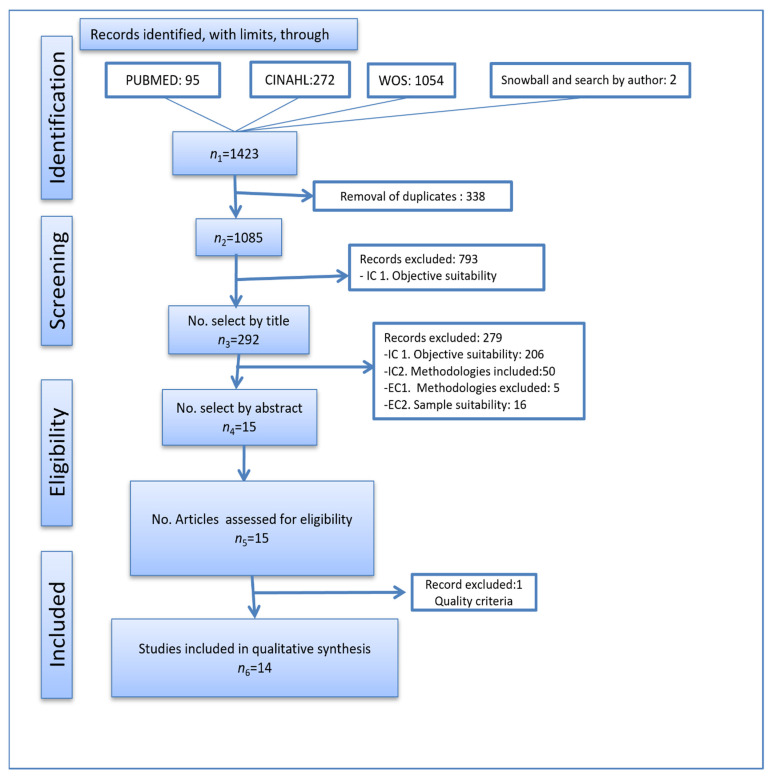
Prisma flow diagram showing the article selection process.

Search outcomes: When the search limits were applied, 1423 articles were found, of which two were identified by searching for key authors using the snowballing technique. Once duplicates were removed, there was a total of 1085 articles remaining. In the review, we discarded studies whose titles did not expressly focus on the perception of nursing competencies. We then proceeded to read the abstracts of the 292 selected titles, applying the established inclusion and exclusion criteria. As a result, 206 were discarded because their objectives were not in line with our research objectives; a further 55 were discarded because they had used a historical method, were letters to the editor, reflection articles, or similar; and a further 16 were discarded because they sought to ascertain nurses’ perceptions of other professional or user groups on the subject. The 15 resulting documents were read in their entirety and were assessed using the MMAT tool. One of them scored poorly and was therefore discarded (see Figure 1).

## 3. Results

### 3.1. Descriptive Analysis

Fourteen articles were included in the review, which, in methodological terms, were three reviews, four quantitative studies, five qualitative studies, and two mixed methods studies. Study samples included young individuals and university students (three); nursing students (five); professionals working with nurses in teams (one); and, the general public users of healthcare facilities (one). Finally, another study took news in the media as a source of information. Table 3 shows a summary of the studies included.

### 3.2. Qualitative Analysis

A number of studies detected widespread ignorance of nursing functions, activities, and roles, and an inability to differentiate them from those exercised by doctors [23,32,33]. However, other studies report that nursing is viewed as a health sciences profession [33] whose primary mission is to care or deliver care [22,28,33,34]. Care is understood as the delivery of help, assistance, and services to patients and sick people, for whom nurses should act as resources during their processes [22,26,34]. The findings on professional competences and social perception are shown below.

#### 3.2.1. Professional Competences Perceived by Society

Some studies have described in great depth the skill sets or groups of competences that society attributes to and/or expects from the nursing profession. We classified their contributions into the five groups of the Tuning framework:

Knowledge and cognitive competences: they include the possession of sound, up-to-date knowledge, a willingness to engage in lifelong learning, and the development of critical thinking skills and emotional intelligence to enable engagement with patients without burnout [26,30,34]. Two studies identified a perception of nurses as less intellectually capable [22] or inclined more to being skilled than to being intelligent [24]. There is limited representation of nursing research and the ability to apply this research evidence to nursing practice. Čukljek [26] reported that the items “Research is vital to nursing as a profession” and “Nurses incorporate research findings in their clinical practice” were viewed as largely irrelevant by their sample, obtaining an average score below agree. In the sample in the study by Albar [34], only 46% answered agree in response to the research profile, and 33% affirmed that nursing practice is based on scientific evidence.

Nursing practice and clinical decision making: nursing practice was mentioned in all the studies reviewed, suggesting that nursing is viewed as a predominantly practical profession [32]. Nursing is understood as a profession that operates primarily in the clinical setting. There is a tendency to perceive the nursing profession as existing primarily within hospitals [28,33,34]. As a result, the hospital setting is the main symbol representing nursing, as it is viewed as the physical space in which most nurses deliver care [28,33]. Other symbols include the uniform or clothing that nurses tend to wear in hospitals [26] and the equipment and instruments they use on a daily basis: stethoscope, gloves, etc. [30]. Nursing students agreed with this relationship between nursing and the hospital as the main working environment. For instance, the first-year nursing students participating in the study by Čukljek [26] explained that nurses care for sick individuals at the hospital. In addition, the sample in the study by Yilmaz [27] saw themselves working as clinical nurses in surgical and paediatric departments, for example, rather than in areas such as education or teaching, administration, and management in the healthcare sector. Other settings, such as home care [24,28] and end-of-life care [22], were rarely mentioned in the studies. Regarding health prevention, promotion, and recovery, Albar [34] reported that 84% of their sample of first-year nursing students agreed that the role of nurses was associated with health prevention, 90% with health promotion, and 94% with recovery. The techniques described include healing wounds, monitoring vital signs, taking samples, administering medication and fluids, taking anthropometric measurements, providing basic care, controlling pain, and working with technology [22,24,28,32,34]. Regarding clinical decision making, the nursing profession is even presented as “providing medical care”, an undeniably vague nuance found in Elmorshedy [25]. In other studies, nursing is viewed as a subordinate profession that depends on, assists, or helps doctors [22,25,29,34], with some alluding to a lack of overall autonomy, and, more specifically, to a lack of decision-making autonomy, and others to a lesser degree of responsibility [22,34]. Despite this being the predominant stance, other studies contradicted perceptions of this subordinate position. In Pierroti [28], nurses are identified as doctors’ assistants due to the 24/7 clinical care provided by the group in comparison with the brief contact between doctors and patients. Girvin [23] detected two conflicting positions; on the one hand, nursing is viewed as an autonomous profession characterised by comprehensive roles, broad knowledge, and high visibility, while on the other hand, nurses are perceived as doctors’ helpers or apprentices. However, Glerean [22] found that nursing is viewed as a profession with a low level of autonomy, despite nurses being perceived as independent professionals. The sample in Čukljek [26] displayed high levels of disagreement with the item “Nurses do not follow physicians’ orders without questions”, echoing Albar [34], in which 36% of the sample of students agreed with the item “Nurses make decisions on care autonomously”. The most extreme contradiction can be found in the study by Sanz Vega [24], where 69.1% of the sample agreed completely or somewhat with the item “Nursing involves functions that do not require a doctor to be present”, and only 2.1% agreed that one of the conditions required to be a nurse was obedience. Meanwhile, 10.7% of the sample disagreed with the item “Nurses only perform activities on a doctor’s orders”, indicating that more than 89% agreed with this statement.

Professional values and the role of the nurse: the professional values with the greatest representation were having a vocation, inclination, drive, or enthusiasm for the profession, and responsibility [24,29,33,34]. On the other hand, other studies included flexibility to change, versatility to take on different roles, and ability to cope with occupational risks, death, and times of maximum demand [22,28,30]. After these, the most represented values were altruism; being humane, possessing human sensibilities, or being humanitarian; respect, tolerance, and open-mindedness; being committed or dedicated to the profession; being hard-working; and self-sacrifice [22,28,29,30,33]. Finally, values with less representation were fellowship and being supportive; professionalism; determination and trustworthiness; solidarity; honesty and compassion; and being disciplined, cautiousness, and meticulousness [22,28,30,33].

Communication and interpersonal competences: these included empathy, active listening, communication skills, friendliness, and ability to establish close interpersonal relationships with patients [22,28,30,34].

Leadership and team working: these include connecting with other members of the multidisciplinary team, being able to engage in collaborative work, efficiency, leadership or charisma, and good time management [29,30]. Competences related to education, teaching, research, and management are only sporadically mentioned among nursing students or any other type of sample and in review studies [24,28,34]. With regard to management, Elmorshedy [25] found that 68.9% of their sample answered disagree to the idea of nurses holding senior management positions, as they viewed them as lacking the necessary training and skills.

#### 3.2.2. Prestige and Value Attached by Society

The sample in Pierroti [28] viewed nursing as an essential, vital profession for improving patients’ health, as nurses are responsible for providing all forms of care, corroborating the findings of a number of studies. In the review by Terry [12], nurses are acknowledged to have a significant impact on the general population. Equally, in Yilmaz [27], 66% of the participants evaluated nurses’ professional status very positively, while only 12.5% evaluated it negatively. The sample in Browne [30] also made a very positive professional evaluation of nurses. The image of nursing observed by Glerean [22] portrays a profession that is highly valued and needed by the population.

More specifically, the sample in Sanz Vega [24] displayed high levels of trust in nurses: 97% of users would welcome nurses into their homes, 76% trusted nurses to apply new techniques, and 50% trusted nurses to prescribe medication, although 71.3% would confirm the prescription with a doctor. In addition, in the study by Čukljek [26], first-year nursing students showed the highest levels of agreement on the following items: “Nurses act as resource persons for individuals with health problems”, and “The service given by nurses is as important as that given by physicians”.

Some studies compared evaluations of different healthcare professionals by nursing students, other healthcare professionals, users, and the general public. With some ambivalence, the sample in Pierroti [28] viewed nurses as equally important as doctors but said that doctors enjoyed greater prestige. In Terry [21], nurses are considered inferior to doctors or with a lower social status. Similarly, Glerean [22] showed that the nursing profession has low levels of recognition or social status. We identified the lowest level of social status in Elmorshedy [25], whose sample, university students and teachers, stated that they would be ashamed to have a nurse in their family.

According to the findings reported by Girvin [23], the nursing profession is sometimes not recommended by school career advisors or family members because it is not viewed as the ideal career. Conversely, in Sanz Vega [24], 73.4% of the respondents viewed the profession as a good occupation that they would recommend to their loved ones.

In consonance with Sánchez-Gras [31], and according to Girvin [23], nurses are generally portrayed in the press as professionals with a secondary role associated with another profession, with little responsibility, autonomy, or decision-making capacity. The nursing profession is conveyed as uninteresting, unchallenging, and lacking in creativity and responsibility, with few opportunities for growth or promotion, with a low academic level, low pay, and low social status. According to these studies, the image conveyed in the media centres around mistakes made by nursing professionals, errors with an impact on patients’ health, negligence, and even crimes committed by nurses [23,31]. In contrast, the sample in Sanz Vega [24] disagreed with the image of nursing portrayed by the media for failing to do justice to the profession’s social status or prestige. According to Girvin [23], websites belonging to official institutions and healthcare facilities reveal the dominance of medicine over the other healthcare professions. Nursing is barely represented: it is not even listed as a service or as one of 10 professions featured, unlike other professions such as medicine [23].

## 4. Discussion

Our findings suggest that nursing is perceived as a healthcare profession whose primary function is to provide care, which is consistent with other studies that also link nurses to care work [8] for the sick in hospital, administering medication [10], or performing patient hygiene [6,35,36]. To this end, society considers it vital, almost a prerequisite, that nurses possess high technical skills, thus perpetuating the classical view of healthcare, nursing care, the nursing profession, and the recipient of care. The social perception of nurses’ knowledge and cognitive competences is in line with American and European structures alike, although nursing research and the application of nursing evidence to nursing practice is considered to be of little to no relevance by society. Even though there is no doubt that nursing research has increased considerably, society still seems to be unaware of nurses’ research competence. This may be related to the image of the clinical nurse, dedicated to caring for sick people in the hospital. Areas such as health promotion, disease prevention, and assisting healthy individuals have not been reflected in society’s perception either, which echoes previous studies [8].

Even communication skills are reduced to interpersonal relations, which are established between nurses and patients in the care setting, ignoring other contexts such as interprofessional relations and communication through mass media, which are included in our profile. The competence to communicate in political contexts and to participate in health policies has not emerged. This is likely to be linked to the dependent role attributed to the profession. The limited involvement and visibility of nurses in these decision-making groups is increasingly evident and vindicated by professionals. In this regard, the International Council of Nurses states that it is critical to ensure that nurses have a say in the development and implementation of healthcare policies in order to ensure that these policies are effective in meeting the real needs of patients, families, and communities around the world. This aspect has been investigated by different authors, such as Benton, Al Maaitah [37]; Rasheed and Younas [38]; and, Cervera-Gasch and Mena-Tudela [39]; who have proposed interesting interventions to include this competence in the nursing colleges. However, in order to achieve this status, society needs to become aware of nurses’ real competences and value them.

Society identifies communicative values and competences, but relates them more to personal attributes than to professional competences. This view is confirmed by studies that identify the following professional values as qualities of nurses: being responsible and skilful [40]; compassionate and kind [13]; kind, patient, and affectionate [41]. This perception favours an out-dated view of the profession, tied to its historical background, thus downplaying the importance of professionalism. In addition, a number of healthcare managers and many nurse educators maintain a vocational style, which can lead to considerable differences between nursing education institutions in terms of the overall vision of nursing [42].

Leadership and team work competences coincide with some of the ideas presented in this review. Society perceives nurses as being able to work collaboratively with other professionals. At the same time, however, society places nurses in an inferior position to other team members. Although the value attributed to the nursing profession is similar to that of the medical profession, it is perceived as a secondary role associated with another profession, lacking the training and skills for team management, with few opportunities for growth or promotion, and with little responsibility, autonomy, or decision-making capacity. This vision is more prominent in certain cultural contexts where this lack of autonomy is understood as obedience, where nurses are perceived as simply following the doctor’s instructions [10,40,43]. The idea of being subordinated to the medical profession also influences the self-concept and professional identity of nurses [4] and their social perception. It is sometimes argued that the social prestige of nursing is poor because it is compared to other professions such as medicine [35,44], highlighting that choosing to study medicine as opposed to nursing is linked to the higher status of medicine [14]. Nevertheless, nurses’ knowledge, skills, and abilities, which underpin the profile defined in the aforementioned programmes, do equip them with the competences to work autonomously and independently. Authors have shown that nursing was considered to be as prestigious as the medical profession, ranking it higher in terms of the trust patients place in them [6]. It is important to note that, according to the annual Gallup report, nurses have been selected for the twentieth year running by the American population as the professionals they trust the most for their honesty and ethics. They were voted for by 81% of the sample, ranking ahead of doctors, teachers, pharmacists, and the military [45]. In addition, our findings suggest that the value that society places on nursing is positive, and it is perceived as an essential profession for improving people’s health.

The limitations of this integrative review include the methodological variability of the studies reviewed, which hindered the comparison of their results, and the wide range of territories covered, which produced conflicting results depending on the majority culture in the region of study.

## 5. Conclusions

The competences attributed by society to nursing professionals do not match the set of competences described in the professional profile of nurses. A lack of knowledge and a partial vision of nursing leave out essential aspects of the profession, such as nurses’ capacity for research and leadership, health policy planning, and health management. These aspects constrain the development of the profession and the creation of a professional identity, and therefore it is essential to make society aware of the real professional competencies of nurses.

This article provides a comparison between the competences defining the nursing profile as per the European and American frameworks, and the skills attributed by society to nurses and how society perceives them. It also provides the ideal framework for comparing reality and perceptions, enabling researchers to identify competence areas that are less known or valued by society in order to be able to address them.

Professional nurses must realise that the solution to the mismatches between the projected image, the perceived image, and reality must come from nurses themselves as a whole and in different contexts, such as faculties, professional associations, scientific associations, etc., focusing on the least known areas, such as research, teaching, and management. In particular, nurses need to take on and project their capacity for leadership to be able to participate fully in the development of health policies and health legislation. To this end, it appears to be essential that nurses follow the ICN guidelines and assume their role as health leaders. Nurses can do this by using traditional media and today’s wide array of social media, embracing and developing their own social media skills, which they could use to make their research results more visible, beyond professional circles. Finally, it is also essential to influence prospective students, e.g., through recruitment open days, and people who influence their choice of career, such as educators, career advisors, and parents, so that they can advise students with knowledge of the reality of the profession’s competences.

## Figures and Tables

**Table 1:** Reference frameworks for the general descriptors of a bachelor’s degree programme in Nursing in Europe and in the United States.

Tuning Educational Structure in Europe. Guidelines and Reference Points for Design and Delivery of Degree Programmes in Nursing				American Association of Colleges of Nursing. The Essentials: Core Competencies for Professional Nursing Education	
Competence Group	Descriptor: Knowledge	Descriptor: Skill	Descriptor: Autonomy and Responsibility	Domains	Descriptor
Knowledge and cognitive competences	Nursing theories, knowledge, and concepts of health, ill health, well-being. The humanities, arts, and sciences necessary to understand human behaviour, bodily functioning, and adaptive responses in different cultures and contexts.	The ability to evaluate evidence and apply this evidence to individual clients, populations, and cultures, so as to deliver effective nursing care in a timely manner.	Being aware of the impact of globalisation, particularly with respect to migration of staff and patients and their health and wellbeing. Knowing how to contribute in the public/civic space during emergency or disaster situations.	1. Knowledge for Nursing Practice	Integration, translation, and application of established and evolving disciplinary nursing knowledge and ways of knowing, as well as knowledge from other disciplines, including a foundation in liberal arts and natural and social sciences.
				4. Scholarship for Nursing Discipline	The generation, synthesis, translation, application, and dissemination of nursing knowledge to improve health and transform health care.
Professional values and the role of the nurse	The professional, moral, ethical and/or legal principles, dilemmas, and issues in day-to-day practice.	The ability to respond appropriately and effectively to professional, moral, ethical, and/or legal dilemmas and issues in day-to-day practice.	Being able to adjust one’s role to respond effectively to population/patient needs within the scope of one’s professional practice and accountability. Being able to challenge current systems to meet population/patient needs where necessary and appropriate.	9. Professionalism	Formation and cultivation of a sustainable professional nursing identity, accountability, perspective, collaborative disposition, and comportment that reflects nursing’s characteristics and values.
Nursing practice and clinical decision making	The principles, concepts, practices, and procedures that underpin the practice and decision making of daily nursing practice.	The ability to make and enact clinical decisions within their scope of practice. The ability to fulfil the scope of practice articulated at the national and European levels. The ability to be a reflective practitioner.	Being able to reflect upon societal and population health and social needs, contributing as appropriate to policy making. Being familiar with cultural competence. Having technical skills that can be utilised in the public space.	2. Person-Centred Care	Person-centred care focuses on the individual within multiple complicated contexts, including family and/or important others. Person-centred care is holistic, individualized, just, respectful, compassionate, coordinated, evidence-based, and developmentally appropriate.
				3. Population Health	Population health spans the healthcare delivery continuum from public health prevention to disease management of populations and describes collaborative activities with both traditional and non-traditional partnerships from affected communities, public health, industry, academia, health care, local government entities, and others for the improvement of equitable population health outcomes.
				5. Quality and Safety	Employment of established and emerging principles of safety and improvement science. Quality and safety, as core values of nursing practice, enhance quality and minimize risk of harm to patients and providers through both system effectiveness and individual performance.
				8. Informatics and Healthcare Technologies	Information and communication technologies and informatics processes are used to provide care, gather data, form information to drive decision making, and support professionals as they expand knowledge and wisdom for practice.
Communication and interpersonal competences	The art and science of communication in a range of circumstances with individuals, groups, and populations in a digital age.	Communicating effectively with diverse peoples and abilities in a range of settings using appropriate media.	Being able to communicate with lay and professional groups with an appreciation of political contexts.	6. Interprofessional Partnerships	Intentional collaboration across professions and with care team members, patients, families, communities, and other stakeholders to optimize care, enhance the healthcare experience, and strengthen outcomes.
Leadership and team work	From the perspective of a new registrant. Theories and models of leadership, followership, management, and teams within health and social care contexts.	Being able to lead and work collaboratively in clinical/healthcare teams. Being able to supervise colleagues and junior staff.	Ability to work interculturally and interprofessionally with both lay and professional groups.	7. Systems-Based Practice	Responding to and leading within complex systems of health care. Nurses effectively and proactively coordinate resources to provide safe, quality, equitable care to diverse populations.
				10. Personal, Professional, and Leadership Development	Participation in activities and self-reflection that foster personal health, resilience, and well-being, lifelong learning, and support the acquisition of nursing expertise and assertion of leadership.

Source: The authors, based on direct quotes from the following reports: Guidelines and Reference Points for the Design and Delivery of Degree Programmes in Nursing [18] (p. 7), and The Essentials: Core Competencies for Professional Nursing Education [17] (pp. 27–54).

**Table 2:** Search strategies.

Term	Search Strategies
1	((((social perception [MeSH Terms])) ^a^ (social perception [MH])) ^b^ OR (social perception [Title/Abstract])) OR (social representation [Title/Abstract])) OR (social image [Title/Abstract]))
2	(((nursing [MeSH Terms]) ^a^ OR (nurse [MH])) ^b^ OR (nurses [MH])) ^b^ OR (nurse [Title/Abstract])) OR (nurses [Title/Abstract]))
3	1 AND 2

^a^ Only for Pubmed. ^b^ Only for Cinahl.

**Table 3:** Summary of the studies included.

Reviews Articles			
Author Year, Country	Aim	Study Design, Sample, Data Collection Method and Analysis	Main Results
Terry, D. 2020 [21] Australia	To examine the psychological constructs that influence male perceptions of nursing as they seek to work in and navigate the profession.	Systematic review and mixed research synthesis *n* = 24 studies Reviewing methods: Sandelwski (2006) Methodological rigour: CASP	Nurses are acknowledged to have a significant impact on the general population but are considered inferior to doctors or considered to have a lower social status. In addition, the nursing profession is viewed as an inferior career associated with other health-related professions, such as medicine.
Glerean, N. 2017 [22] Finland	To describe young peoples’ perceptions of the nursing profession and to identify factors influencing them	Integrative literature review *n* = 8 studies Reviewing methods: Whittemore and Knafl (2005) Methodological rigour: JBI quality appraisal tools	Nurses’ work includes patient care, helping others, working with technology, being in contact with illness, death, and biological materials. Nurses’ working conditions are hard, stressful, and busy, with a high risk of injury. Nurses are needed and respected. Nurses are not independent and cannot make decisions for themselves. Nurses have the role of assistants to doctors. Nurses have job security and salary, but their opportunities in management and leadership are restricted. Nursing is a meaningful job and at the same time has little prestige and low status in society. Nurses are considered to be kind, caring and helpful, physically strong and with good social skills, as well as sympathetic, reliable and open-minded, hard-working, determined to endure the sight of blood, and able to cope with death, although less intellectually capable.
Girvin, J. 2016 [23] United Kingdom	To investigate the current public understanding and perceptions of nursing	Systematic review and narrative synthesis *n* = 21 studies Narrative analysis and narrative data methods Methodological rigour: MMAT	They have detected widespread ignorance of nursing functions, activities, and roles, and an inability to differentiate them from those exercised by doctors. Nursing was viewed as an autonomous profession characterised by comprehensive roles, broad knowledge, and high visibility, while nurses are perceived as doctors’ helpers or apprentices. The nursing profession was considered to be of low-image, not interesting or “stretching”, a profession that lacked challenge, creativity, and responsibility, and which presented few opportunities for promotion, comparable to office work or hairdressing. Nursing is under-represented on health service web pages. In media, nurses were generally portrayed as professionals with a secondary role. Nursing was not viewed as an ideal career by school career advisors, and few family members would recommend nursing as a career to their relatives. The public rarely identified nursing in leadership roles.
**Quantitative Studies**			
**Author** **Year, Country**	**Aim**	**Study Design, Sample,** **Data Collection Method and Analysis**	**Main Results**
Sanz-Vega, C. 2020 [24] Spain	To ascertain the social image of nursing among the Asturian population	Quantitative multicentre descriptive study *n* = 335 participants Self-report questionnaire Quantitative and statistical analyses	Nursing is viewed as a predominantly practical profession, operating mainly in clinical settings, both in hospitals and at home. Nurses provide basic care, control pain, and administer medication and fluids. Nurses must have an inclination, drive, or enthusiasm for the profession, and be more skilled than intelligent. Other areas of nursing work, which were less well represented, were health prevention, promotion, recovery, and education. The sample showed high levels of trust in nurses: 97% of users would welcome nurses in their home. The evaluations of different healthcare professions were compared, and nursing came second only to medicine. A total of 69.1% strongly agreed or agreed with the item, “Nursing involves functions that do not require a doctor to be present”, but only 10.7% disagreed with the item, “Nurses only perform activities on doctor’s orders”.
Elmorshedy H. 2020 [25] Saudi Arabia	To explore the level of community awareness and public image of the nursing profession in Saudi Arabia	Quantitative, cross-sectional study *n* = 502 university students enrolled in non-health college Ad hoc, self-report survey Quantitative statistical analysis	The nursing profession is viewed as “providing medical care”, and nurses as assistants to doctors. A total of 68.9% of the sample answered disagree to the idea of nurses holding senior management positions, as they viewed them as lacking the necessary training and skills. Some would be ashamed if they had a nurse in their family.
Čukljek, S. 2017 [26] Croatia	To determine the attitudes of nursing students towards nursing	Quantitative study with a pre-post survey *n* = 115 first-year nursing student Nursing Image Questionnaire Likert scale (1–5) Quantitative and descriptive statistical analyses	Nurses act as resource persons for individuals with health problems 4.50. Nurses are patients’ advocates 3.70. Nurses with completed undergraduate and graduate nursing studies significantly contribute to patient care 4.32. Nurses integrate health teaching into their practice 4.26. It takes intelligence to be a nurse 3.72. Nurses in general are kind, compassionate human beings 3.51. Nurses consistently update their practices in relation to current health trends 3.52. The service given by nurses is as important as that given by physicians 4.40. Nurses are capable of independent practice 3.91. Nurses incorporate research findings into their clinical practice 3.62. The major goal of nursing research is to improve patient care. 4.17. Nurses should not wear the blue uniform in order to be identified 2.34.
Yilmaz, A. 2016 [27] Turkey	To investigate the effect of career-planning events for nursing students on their conceptualisations of the nursing profession and their career plans	Quantitative experimental study with pre-test and post-test *n* = 129 first-year nursing students Perception of Nursing Profession Scale Quantitative and descriptive statistical analysis	A total of 40% of the participants reported that they would like to work as specialist nurses or nursing staff at any clinic, 23.8% as academics, and 16.2% as administrative nurses. Home care emerged as another working environment. The participants preferred to work in fields such as infection, paediatrics, gynaecology–obstetrics, and the operating theatre in public hospitals.
**Qualitative Studies**			
**Author** **Year, Country**	**Aim**	**Study Design, Sample,** **Data Collection Method, and Analysis**	**Main Results**
Pierroti, V. 2020 [28] Brazil	To understand high school students’ perceptions of nurses’ images and roles in society based on nursing knowledge patterns	Qualitative study *n* = 8 interdisciplinary higher education students Semi-structured interviews Phenomenological qualitative analysis	Participants attributed positive personal characteristics to nurses, such as being caring, careful, responsible, patient, and dedicated to the profession. Nurses are defined as the professionals who first welcome patients in health services, which is essential and crucial. Nurses should be able to deal with people who do not collaborate, know the right dosage of each medication, and be preventative and meticulous. Nurses’ image was predominantly associated with the hospital setting, with an emphasis on technical activities such as body hygiene, medications, etc. The interviewees felt that nurses are physicians’ assistants and that physicians have greater prestige. Nurses spend more time with the patient, 24 h a day. Nurses take a more humane approach to care and interact more with the person being cared for.
Çetinkaya, A. 2019 [29] Turkey	To determine how the concept of nursing was perceived by intern doctors working at a medical faculty hospital	Qualitative study *n* = 54 intern doctors Conceptual analyses of nursing using the Word Association Test Words frequency analysis	Nursing is viewed as a subordinate profession whose aim is to helps doctors. Altruism, devotion, and self-sacrifice are some professional identity terms associated with nurses. Nurses must be competent practitioners.
Browne, C. 2018 [30] Australia	To develop a greater understanding of the perceptions that students, about to embark on their undergraduate nursing degree, had of the nursing profession	Qualitative study *n* = 110 first-year nursing students Creation of drawings and concept maps to define the nursing profession in small groups by consensus Thematic analysis	The role of the nurse as a carer or as caring came through strongly. Nursing is associated with equipment (stethoscopes, gloves, etc.). Their sample rated the nursing profession very positively. Nurses needed to work and communicate with other members of the multidisciplinary team and with patients from diverse backgrounds. Nurses must be able to provide care with compassion, efficiency, leadership, respect, and tolerance. They must have flexibility to change, versatility to take on different roles, and good time management. Being a nurse is attached to attributes such as team work, collaborative work, strong communication, being a good listener, and having good bedside manners. Competent nurse practitioners are knowledgeable, critical thinkers, and lifelong learners.
Sánchez-Gras, S. 2017 [31] Spain	To present an exhaustive critical analysis of the treatment received by the nursing profession and nurses in the written press	Qualitative analytical study based on grey literature *n* = 235 news articles News articles published in regional and national written press outlets containing the term “nursing” Qualitative content analysis	Nursing is conveyed as an uninteresting profession with few opportunities for growth or promotion. Nurses are generally portrayed as professionals with a secondary role associated with another profession and with little autonomy. There are lots of news stories about mistakes made by nursing professionals and errors with an impact on patients’ health.
Crawford, R. 2016 [32] New Zealand	To understand the discourse amongst a range of health professional students, including nursing, talking about nurses and nursing	Qualitative descriptive study *n* = 32 students on an interprofessional immersion programme 9 focus groups Analysis by comparing datum with datum until recurrent themes emerged	Nursing was described as “hands-on stuff”, and it is important to “do nursing”. Nursing is viewed as a predominantly practical profession. Vague encapsulation of the profession and its skills. There are references to some psychomotor skills. Nurses organise health checks, weigh patients, take blood pressure and heart rate, and run blood sugar tests.
**Mixed Methods Studies**			
**Author** **Year, Country**	**Aim**	**Study Design, Sample,** **Data Collection Method, and Analysis**	**Main Results**
Bastias 2020 [33] Argentina	To explore and compare social representations of nurses held by incoming and outgoing nursing students in a technical nursing programme	Qualitative and quantitative descriptive study *n* = 104 first-year nursing students The word association technique for the term “nurse” Prototypical analysis of social representations from a structural perspective	Nursing is a health sciences profession whose primary mission is to care or deliver care in the hospital. There is certain ignorance of nursing functions, activities, and roles, and an inability to differentiate them from those exercised by doctors. Nurses must have a vocation or inclination, they must be humane, possess human sensitivities, and be able to engage in collaborative work. They must display professionalism, honesty, and compassion. Other terms, such as injection and hand washing, also emerged.
Albar, M.J. 2016 [34] Spain	To identify perceptions of the nursing professional identity among first- and fourth-year undergraduate nursing students	Qualitative and quantitative descriptive sectional study *n* = 50 first-year nursing students Questionnaire, by expert consensus, with quantitative scales and open questions Descriptive statistics and content analysis	Nursing is defined as a health sciences profession whose primary mission is to care or deliver care. This care is understood as the delivery of help and assistance to patients and sick people in the hospital. Nurses must be able to listen actively, must be able to establish close interpersonal relationships with patients, possess solid, up-to-date knowledge, and be willing to engage in lifelong learning. Additionally, nurses must have a vocation and responsibilities and be able to treat wounds and monitor vital signs. Other working environments, such as health prevention (84% agreed), health promotion (90% agreed), recovery (94% agreed), and research (46% agreed) emerged. Health education, teaching, and management did not emerge. Nursing is an autonomous profession: 84% disagreed or were undecided. Nursing is a profession that depends on medicine: 52% agreed. Nurses can make decisions autonomously: 36% agreed.

## Data Availability

Not applicable.

## References

[1] Fargemoen M.S. (1997). Professional identity: Values embedded un meanningful nursing practice. J. Adv. Nurs..

[2] Celma Vicente M. (2007). Cultura Organizacional y Desarrollo Profesional de las Enfermeras. Ph.D. Thesis.

[3] Pimentel M.H., Pereira F.A., Pereira da Mata M.A. (2011). La construcción de la identidad social y profesional de una profesión femenina: Enfermería. Prism. Soc..

[4] Hoeve Y., Jansen G., Roodbol P. (2014). The nursing profession: Public image, self-concept and professional identity. J. Adv. Nurs..

[5] Vega Angarita O.M. (2006). Estructura del conocimientos contemporaneo de enfermería. Rev. Cienc. Cuid..

[6] Woods-Giscombe C.L., Johnson Rowsey P., Kneipp S., Lackey C., Bravo L. (2020). Student perspectives on recruiting underrepresented ethnic minority students to nursing: Enhancing outreach, engaging family, and correcting misconceptions. J. Prof. Nurs..

[7] Rasmussen P., Henderson A., McCallum J., Andrew N. (2021). Professional identity in nursing: A mixed method research study. Nurse Educ. Pract..

[8] Teresa-Morales C., González-Sanz J.D., Rodríguez-Pérez M. (2021). Components of the nursing role as perceived by first-year nursing students. Nurse Educ. Today.

[9] Jamieson I., Harding T., Withington J., Hudson D. (2019). Men entering nursing: Has anything changed?. Nurs. Prax. N. Z..

[10] Chen Y., Zhang Y., Jin R. (2020). Professional Identity of Male Nursing Students in 3-Year Colleges and Junior Male Nurses in China. Am. J. Mens Health.

[11] López-Verdugo M., Ponce-Blandón J.A., López-Narbona F.J., Romero-Castillo R., Guerra-Martín M.D. (2021). Social Image of Nursing. An Integrative Review about a Yet Unknown Profession. Nurs. Rep..

[12] Yang C.-I., Yu H.-Y., Chin Y.-F., Lee L.-H. (2017). There is nothing wrong with being a nurse: The experiences of male nursing students in Taiwan. Jpn. J. Nurs. Sci..

[13] Liu H.Y., Li Y.L. (2017). Crossing the gender boundaries: The gender experiences of male nursing students in initial nursing clinical practice in Taiwan. Nurse Educ. Today.

[14] Alexander R.K., Diefenbeck C. (2020). Challenging stereotypes: A glimpse into nursing’s difficulty recruiting African Americans. J. Prof. Nurs..

[15] Norman K.M. (2015). The image of community nursing: Implications for future student nurse recruitment. Br. J. Community Nurs..

[16] Crisp N. (2018). Nursing-the wave of the future. BMJ.

[17] American Association of Colleges of Nursing (2021). The Essentials: Core Competencies for Professional Nursing Education. https://www.aacnnursing.org/Portals/42/AcademicNursing/pdf/Essentials-2021.pdf.

[18] European Union (2018). Tuning Educational Structures in Europe. Guidelines and References Points for the Design and Delivery of Degree Programmes in Nursing.

[19] Whittemore R., Knafl K. (2005). The integrative review: Updated methodology. J. Adv. Nurs..

[20] Hong Q., Pluye P., Fábregues S., Bartlett G., Boardman F., Cargo M., Pierre D., Marie-Pierre G., Frances G., Belindah N. (2018). Mixed Methods Appraisal Tool (MMAT), version 2018. http://mixedmethodsappraisaltoolpublic.pbworks.com/w/file/fetch/127916259/MMAT_2018_criteria-manual_2018-08-01_ENG.pdf.

[21] Terry D., Peck B., Carden C., Perkins A.J., Smith A. (2020). Traversing the Funambulist’s Fine Line between Nursing and Male Identity: A Systematic Review of the Factors that Influence Men as They Seek to Navigate the Nursing Profession. Eur. J. Investig. Health Psychol. Educ..

[22] Glerean N., Hupli M., Talman K., Haavisto E. (2017). Young peoples’ perceptions of the nursing profession: An integrative review. Nurse Educ. Today.

[23] Girvin J., Jackson D., Hutchinson M. (2016). Contemporary public perceptions of nursing: A systematic review and narrative synthesis of the international research evidence. J. Nurs. Manag..

[24] Sanz Vega C.M., Martínez Espinosa A., Longo Alonso C., Charro Alonso S., Antón Martínez G., Losada Riesgo V.C. (2020). Una fotografía de la imagen social de la Enfermería. RqR Enfermería Comunitaria Rev. SEAPA.

[25] Elmorshedy H., AlAmrani A., Hassan M.H.A., Fayed A., Albrecht S.A. (2020). Contemporary public image of the nursing profession in Saudi Arabia. BMC Nurs..

[26] Čukljek S., Jureša V., Grgas Bile C., Režek B. (2017). Changes in Nursing Students’ Attitudes Towards Nursing During Undergraduate Study. Acta Clin. Croat.

[27] Yilmaz A.A., Ilce A., Can Cicek S., Yuzden G.E., Yigit U. (2016). The effect of a career activity on the students’ perception of the nursing profession and their career plan: A single-group experimental study. Nurse Educ. Today.

[28] Pierroti V.W., Guirardello E.d.B., Toledo V.P. (2020). Nursing knowledge patterns: Nurses’ image and role in society perceived by students. Rev. Bras. Enferm..

[29] Çetinkaya A., Rahman S., Elbi H., Altan S. (2019). Perceptions of intern physicians about nursing profession: A qualitative research. Cukurova Med. J..

[30] Browne C., Wall P., Batt S., Bennett R. (2018). Understanding perceptions of nursing professional identity in students entering an Australian undergraduate nursing degree. Nurse Educ. Pract..

[31] Sánchez-Gras S. (2017). Imagen de la enfermería a través de la prensa escrita ¿necesitamos visibilizar los cuidados enfermeros?. Cult. Cuid..

[32] Crawford R.M., Gallagher P., Harding T., McKinlay E.M., Pullon S.R. (2016). Interprofessional undergraduate students talk about nurses and nursing: A qualitative study. Nurse Educ. Today.

[33] Bastias F., Giménez I., Fabaro P., Ariza J., Caño-Nappa M.J. (2020). Social representations of nurses. Differences between incoming and outgoing Nursing students. Investig. Educ. Enferm..

[34] Albar M.J., Sivianes-Fernández M. (2016). Percepción de la identidad profesional de la enfermería en el alumnado de grado. Enfermería Clínica.

[35] Liaw S.Y., Wu L.T., Holroyd E., Wang W., Lopez V., Lim S., Chow Y.L. (2016). Why not nursing? Factors influencing healthcare career choice among Singaporean students. Int. Nurs. Rev..

[36] Marcinowicz L., Owlasiuk A., Perkowska E. (2016). Exploring the ways experienced nurses in Poland view their profession: A focus group study. Int. Nurs. Rev..

[37] Benton D.C., Al Maaitah R., Gharaibeh M. (2017). An integrative review of pursing policy and political competence. Int. Nurs. Rev..

[38] Rasheed S.P., Younas A., Mehdi F. (2020). Challenges, Extent of Involvement, and the Impact of Nurses’ Involvement in Politics and Policy Making in in Last Two Decades: An Integrative Review. J. Nurs. Scholarsh..

[39] Cervera-Gasch Á., Mena-Tudela D., Castro-Sánchez E., Santillan-Garcia A., Andreu-Pejó L., González-Chordá V.M. (2022). Necessary political competences for nurses from the perception of the student body: Cross-sectional study in Spain. Nurse Educ. Today.

[40] Kluczyńska U. (2017). Motives for choosing and resigning from nursing by men and the definition of masculinity: A qualitative study. J. Adv. Nurs..

[41] Teresa Morales C., Feria Lorenzo D.J., Rodríguez Pérez M. (2019). Imagen social y valoración de la profesión enfermera para el alumnado del Grado de Enfermería. Temperamentvm.

[42] Findlow S. (2012). Higher education change and professional-academic identity in newly ‘academic’ disciplines: The case of nurse education. High. Educ..

[43] Cheng M.L., Tseng Y.H., Hodges E., Chou F.H. (2018). Lived Experiences of Novice Male Nurses in Taiwan. J. Transcult. Nurs..

[44] Kämmer J.E., Ewers M. (2021). Stereotypes of experienced health professionals in an interprofessional context: Results from a cross-sectional survey in Germany. J. Interprof. Care.

[45] Gallup (2021). Gallup’s Annual Rating of the Honesty and Ethics of Various Professions. https://news.gallup.com/poll/388649/military-brass-judges-among-professions-new-image-lows.aspx.

